# An Efficient *Agrobacterium* Mediated Transformation of Pineapple with GFP-Tagged Protein Allows Easy, Non-Destructive Screening of Transgenic Pineapple Plants

**DOI:** 10.3390/biom9100617

**Published:** 2019-10-17

**Authors:** S. V. G. N. Priyadarshani, Hanyang Cai, Qiao Zhou, Yanhui Liu, Yan Cheng, Junjie Xiong, Dikoko Lesego Patson, Shijiang Cao, Heming Zhao, Yuan Qin

**Affiliations:** 1 Key Lab of Genetics, Breeding and Multiple Utilization of Crops, Ministry of Education, Fujian Provincial Key Laboratory of Haixia Applied Plant Systems Biology, State Key Laboratory of Ecological Pest Control for Fujian and Taiwan Crops, Center for Genomics and Biotechnology, College of Agriculture, Fujian Agriculture and Forestry University, Fuzhou 350002, China; 2 College of Life Sciences, Fujian Agriculture and Forestry University, Fuzhou 350002, China; 3 College of Forestry, Fujian Agriculture and Forestry University, Fuzhou 350002, China; 4 State Key Laboratory for Conservation and Utilization of Subtropical Agro-Bioresources, Guangxi Key Lab of Sugarcane Biology, College of Agriculture, Guangxi University, Nanning 530004, Guangxi, China

**Keywords:** pineapple, *Agrobacterium*, transformation, GFP-tagged protein, *WRKY* genes

## Abstract

Quite a few studies have been conducted to improve the *Agrobacterium*-mediated transformation of pineapple, which is the second most important commercial tropical fruit crop worldwide. However, pineapple transformation remains challenging, due to technical difficulties, the lengthy regeneration process, and a high labor requirement. There have not been any studies specifically addressing the introduction of GFP-tagged genes into pineapples through *Agrobacterium*-mediated transformation, which would enable easy, non-destructive expression detection. It would also allow expression localization at the organelle level, which is not possible with GUS a reporter gene that encodes β-glucuronidase or a herbicide resistance reporter gene. Here, we report a method for the introduction of GFP-tagged genes into pineapples through *Agrobacterium*-mediated transformation. We used embryonic calli for transformation, and plants were regenerated through somatic embryogenesis. In this study, we optimized the incubation time for *Agrobacterium* infection, the co-cultivation time, the hygromycin concentration for selection, and the callus growth conditions after selection. Our strategy reduced the time required to obtain transgenic plants from 7.6 months to 6.1 months. The expression of GFP-tagged *AcWRKY28* was observed in the nuclei of transgenic pineapple root cells. This method allows easy, non-destructive expression detection of transgenic constructs at the organelle level. These findings on pineapple transformation will help accelerate pineapple molecular breeding efforts to introduce new desirable traits.

## 1. Introduction

Pineapple is an economically important tropical fruit crop, and it is used as a fiber crop and source of the valuable pharmaceutical enzyme bromelain [1,2]. There have been extensive efforts to develop new varieties through hybridization of this self-incompatible species, but only a few attempts have been successful. Conventional breeding is difficult due to the high level of genomic heterozygosity and noticeable genome instability in pineapple [3].

As with traditional breeding, *Agrobacterium*-mediated transformation is challenging in pineapple, and studies aimed at introducing new desired traits to improve pineapple quality have been limited. Nevertheless, some important traits have been introduced into pineapples through *Agrobacterium*-mediated transformation and biolistic transformation. Examples include the introduction of genes to control blackheart disease, which is a physiological disorder of pineapple fruit occurring, due to low temperature and low light conditions during fruit development [4], genes for herbicide tolerance [5], and genetic changes to delay natural flowering [6]. Efforts have also been made to improve the transformation process by reducing the time required for plant regeneration via direct regeneration of adventitious buds from infected stem disks and leaf bases [7]. Despite these advances, pineapple transformation is not as fully developed as transformation in other crops, such as rice, due in part to difficulties in the time-consuming regeneration process. Moreover, transformation efficiency has been shown to be as low as 0.12–2.26% [7]. 

A few years back, bacterial β-glucoronidase, commonly referred to as GUS gene, was used as a reporter gene for molecular transformation facilitating as a tool for the analysis of plant gene expression [8]. However, GUS has its limitations and drawbacks as a reporter gene. Among those, diffusion of the staining patterns misleads specificity of expression and GUS treatment is destructive [9,10]. Therefore, the aim of the present study was to develop an efficient transformation strategy through process optimization, particularly for the expression of GFP-tagged genes to allow easy detection of protein expression levels and localization at the organelle level in vivo. 

In this study, we focused on the transformation of a construct containing a *WRKY* gene because *WRKY* genes are known to regulate plant growth and development and to confer tolerance to abiotic stresses including salinity, drought, heat, cold, and wounding [11,12]. WRKY transcription factors (TFs) are numerous in plants; for example, there are 109 in rice and 74 in *Arabidopsis* [12]. In pineapple, there are 54 WRKY TFs, and they have been reported to be responsive to different stress conditions [13]. *AtWRKY50*, which is orthologous of *AcWRKY28* considered as the stress responsive gene [14]. The findings of Gao et al. (2011) also indicated that WRKY46, WRKY50, WRKY51, WRKY53, WRKY60, and WRKY70 proteins might regulate the expression of defense genes. 

Among the 54 *WRKY* genes in the pineapple genome, we selected *AcWRKY28*, an ortholog of *Arabidopsis AtWRKY51* [15], for our analysis of *Agrobacterium*-mediated transformation in pineapple. Specifically, we optimized the conditions for the transformation of *AcWRKY28* with *GFP* as the reporter gene, to enable easy detection and localization of the expressed protein at the organelle level. 

## 2. Materials and Methods 

### 2.1. Bioinformatics Analysis of AcWRKY28

Homologous protein sequences were downloaded from NCBI (www.ncbi.nlm.nih.gov) for sequence comparisons and phylogenetic analysis of *AcWRKY28*. The exon-intron gene structures were determined using the Gene Structure Display Server (http://gsds.cbi.pku.edu.cn) by comparing the full-length predicted coding sequences (CDS) with the corresponding genomic DNA sequences. MEGA 6.0 software was used to generate an unrooted phylogenetic tree based on a neighbor-joining (NJ) procedure with the following parameters: Poisson model, pairwise gap deletion, and 1000 bootstraps [16]. Multiple sequence alignment was performed on the amino acid sequences using ClustalW software, and the genomic location of *AcWRKY28* was analyzed.

### 2.2. Plant Material Preparation

Embryonic calli and micropropagated plants of pineapple variety Tainong 11 were propagated as described by Priyadarshani et al. (2018). All cultures were maintained under 3000 lx light intensity and a day/night cycle of 8/16 h at 25 ± 2 °C in a controlled environment.

### 2.3. Vector Construction and Agrobacterium Culture Preparation for Transformation

The *p35S:GFP-WRKY28* construct was generated by amplifying a 609 bp sequence from pineapple cDNA using the primers WRKY28F/R.

*WRKY28-F*: CACCATGGCGGCTTTACACGCCGCAG

*WRKY28-R*: TCACGATGAACCTGGAGGGAT

The PCR product was cloned into the pENTR/D-TOPO vector (Invitrogen). pENTR/D-TOPO clones were recombined into the destination vector pGWB506 using LR Clonase II (Invitrogen). After sequencing, the confirmed plasmid was transformed into *Agrobacterium*.

A single *Agrobacterium* colony containing binary vector was cultured overnight in 5 mL LB medium containing 50 mg/L rifampicin and 50 mg/L streptomycin with shaking at 150 rpm at 28 °C. The bacterial culture was diluted to a volume of 50 mL LB medium containing 50 mg/L rifampicin, 50 mg/L spectinomycin, and 200 µmol/L acetosyringone (AS) then incubated with shaking at 150 rpm until the OD_600_ value reached 0.6–0.8. The bacteria were harvested by centrifuging at 4000 rpm for 10 min at 25 °C. The supernatant was discarded, and the pellet was resuspended in AAM liquid medium supplemented with 200 µmol/L acetosyringone (Table 1). 

### 2.4. Pineapple Transformation

Well-grown healthy, green embryonic calli were used for transformation. Healthy calli were generated using the method described in Priyadarshani et al. (2018). Calli were cut to a size of 0.5–1.0 cm, directly placed in *Agrobacterium* suspension, then shaken mildly (30–60 rpm) for different time intervals to optimize the incubation time for proper infection. Batches of 100 calli pieces in three replicates were used in each experiment. After shaking, the liquid was poured out, and the calli were blotted on sterile tissue and air-dried for 30 min under aseptic conditions. The calli were transferred into co-cultivation medium supplemented with 200 µmol/L acetosyringone (Table 1) and incubated at 25 °C for 3–4 days in the dark. After three days, the calli were transferred to the proliferation medium containing 200 mg/L carbenicillin (Table 1) to prevent bacterial growth and allow calli proliferation.

After 4–5 weeks, when the calli had grown sufficiently large, they were transferred to selection medium containing 20 mg/L hygromycin. After three weeks in the selection medium, the growing calli were transferred to non-selection medium (hygromycin-free proliferation medium). This selection was performed three times to minimize the multiplication of non-transformed calli. Putatively positive calli were allowed to grow in the proliferation medium to obtain plants. Plants were further selected in medium containing 20 mg/L hygromycin, and the selected plants were transferred to rooting medium for further growth.

### 2.5. Optimization of Factors Influencing Transformation Efficiency

Transformation efficiency is affected by several factors. We optimized the shaking duration of calli in *Agrobacterium* culture for infection, the co-cultivation duration, and the acetosyringone concentration in the co-cultivation medium. All these experiments were performed with at least three independent replicates, each consisting of 100 samples.

### 2.6. Optimization of Hygromycin Concentration for the Selection of Putative Transgenic Lines

The calli were transferred to selection medium supplemented with 10–50 mg/L hygromycin. The survival rate of calli was statistically analyzed after one month. All these experiments were performed with at least three independent replicates, each consisting of 100 samples.

### 2.7. Effect of Different Growth Conditions on Callus Growth after Selection

Calli after the third hygromycin selection was transferred to two different final non-selection media for callus and shoot growth. We used hormone-free liquid MS medium and 0.2 mg/L NAA solid MS medium supplemented with 30 g/L sugar. Measurements were obtained after 2 weeks of inoculation.

### 2.8. Data Collection and Statistical Analysis

Surviving calli were counted after four weeks, and the survival percentage was calculated to optimize different conditions for transformation. A completely randomized design was used at a 5% significance level, and Analysis of Variance (ANOVA) was performed using MINITAB 16 statistical analysis software.

### 2.9. DNA Extraction and PCR Analysis

Genomic DNA was isolated from the leaves of putative positive plants using a slightly modified CTAB method [17] as follows. Leaf samples were collected into 2.0 mL Eppendorf tubes, which were immediately placed in liquid N_2_. Samples were ground to a powder using a plastic pestle. A 500 μL volume of CTAB solution was added, followed by incubation at 65 °C for 1 h and centrifugation at 12,000 rpm for 10 min. The supernatant was transferred to a new 2.0 mL Eppendorf tube. An equivalent volume of 24:1 chloroform:isopropanol was added to the collected supernatant, and the tubes were inverted to mix the solution gently. Samples were centrifuged at 12,000 rpm for 10 min to separate the aqueous layer with dissolved DNA. The aqueous upper layer was transferred to a new 1.5 mL Eppendorf tube, and DNA was precipitated by adding cold isopropanol (1:0.6). The samples were incubated for 30 min at −20 °C then centrifuged at 12,000 rpm for 10 min to precipitate DNA. The supernatant was discarded, and the precipitate was washed with 75% alcohol and centrifuged at 12,000 rpm for 10 min. The alcohol was discarded, and the precipitate was dried to remove all ethanol. Finally, the DNA was dissolved in 30 µL of sterilized distilled water. 

The presence of the *p35S:GFP-WRKY28* construct was confirmed by PCR analysis using specific primers for GFP and the *AcWRKY28* gene (*GFP-F:* TGCAGATGAACTTCAGGGTCAGC, *WRKY28-R*: TCACGATGAACCTGGAGG GAT). The PCR reaction was performed using the 2X Taq Master Mix Kit (Vazyme) according to the manufacturer’s instructions. Table 2 shows the reaction mixture composition. The expected size of the *GFP-AcWRKY28* product was 1200 bp, and the reaction conditions were as follows: Initial denaturation at 95 °C for 3 min; 34 cycles of 95 °C for 30 s, 62 °C for 30 s, and 72 °C for 80 s; and a final extension at 72 °C for 10 min. The PCR product was observed on a 1% agarose gel.

### 2.10. Microscopy

The roots of transformed plants were observed under a confocal laser scanning microscope (Leica TCS SP8X). The excitation wavelengths were set to 488–507 nm for GFP and 659–749 nm for auto-fluorescence. 

## 3. Results

### 3.1. Sequence Analysis

**Figure 1:**
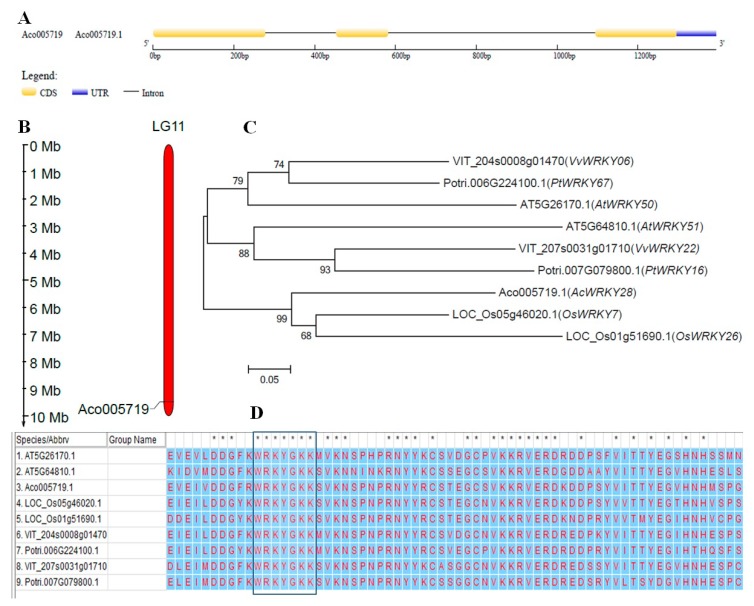
Bioinformatics analysis of *AcWRKY28*. (**A**) Gene structure of *AcWRKY28*. (**B**) Chromosomal location of *AcWRKY28*. (**C**) Phylogenetic relationship between *AcWRKY28* and other WRKY family members from different plant species. The unrooted phylogenetic tree was generated using MEGA 6.0 software with neighbor-joining procedure following mentioned parameters: Poisson model, pairwise gap deletion and 1000 bootstraps. Bar = 0.05 indicates the distance scale. (**D**) Multiple sequence alignment of homologous sequences from different plant species.

Among the 54 *AcWRKY* genes, we focused on *AcWRKY28* (*Aco005719.1*) for the analysis and transformation. The intron, exon, and UTR arrangement of the gene are shown in Figure 1A. The *AcWRKY28* open reading frame (ORF) length is 609 bp, and further analysis revealed that the gene is located on chromosome 11 (Figure 1B). A phylogenetic tree was constructed to investigate the relationship between *AcWRKY28* and its homologous sequences, and *AcWRKY28* was found to be closely related to *AT5G64810.1* (*AtWRKY51*) (Figure 1C). Fifty of the pineapple AcWRKY proteins have a highly conserved sequence WRKYGQK [13]. However, AcWRKY28 has the WRKYGKK sequence (Figure 1D).

### 3.2. Optimization of Incubation Time for Infection, Co-Cultivation Duration, and Acetosyringone Concentration in the Co-Cultivation Medium

To optimize factors affecting transformation efficiency, we tested the shaking duration of calli in *Agrobacterium* culture for infection, the co-cultivation duration, and the acetosyringone concentration in the co-cultivation medium. As shown in Table 3, an incubation time within the range of 1–6 h was optimal for the infection period to get infected calli without overgrowth of bacteria and death of infected calli when cultivated in the co-cultivation medium.

For the acetosyringone concentration analysis, there was no significant difference in putative transformant selection between 200 µM and 400 µM acetosyringone in the co-cultivation medium. We, therefore, selected 200 µM as the optimal acetosyringone concentration for the co-cultivation medium.

**Figure 2:**
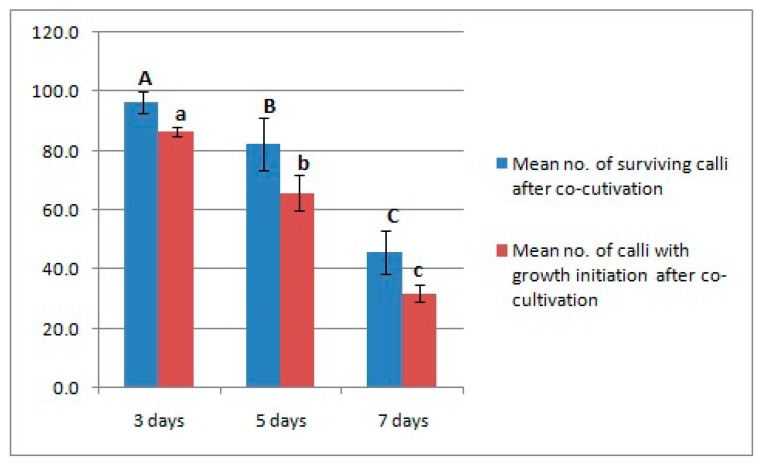
A number of surviving calli after different durations of co-cultivation. Error bars indicate standard deviation (SD). Different letters represent statistically significant differences at *p* = 0.05.

Because the duration of co-cultivation may affect the efficiency of *Agrobacterium* infection of plant material and transformation efficiency overall, we tested the effects of different co-cultivation periods (3, 5, and 7 days). We observed that the number of surviving calli without *Agrobacterium* overgrowth was significantly higher after three days of co-cultivation. Furthermore, callus growth was also significantly higher after three days, with a reduced number of calli exhibiting growth after a co-cultivation period of seven days (Figure 2). We, therefore, selected three days as the optimal co-cultivation duration for transformation.

### 3.3. Optimization of Hygromycin Concentration for the Selection of Transgenic Lines

**Figure 3:**
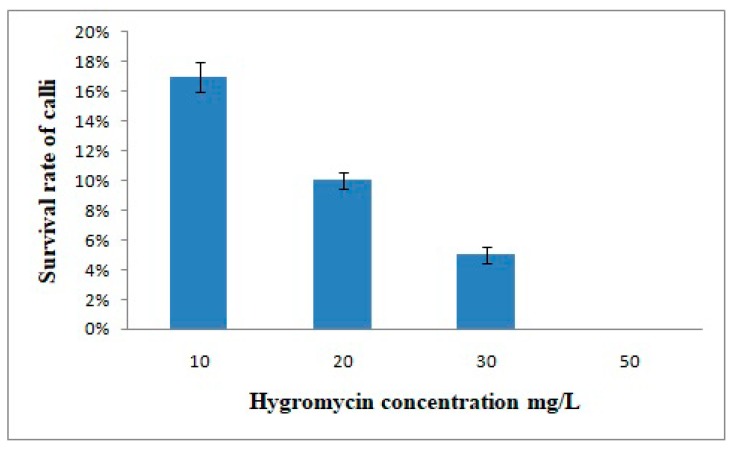
Effect of different hygromycin concentrations on callus survival. Error bars indicate Standard Error (SE).

**Figure 4:**
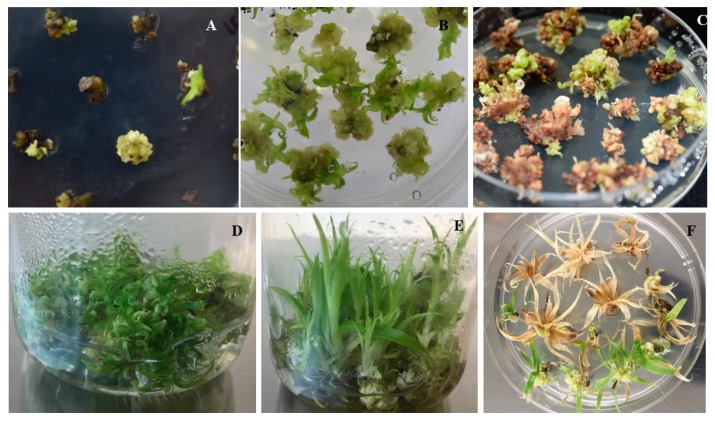
Different stages of the pineapple transformation process. (**A**) Putative transformants are growing on the first selection medium. (**B**) Calli growing on non-selection medium. (**C**) Calli growing on the second selection medium. (**D**) Putative transformed calli were grown on solid medium for two months. (**E**) Putative transformed calli grew into plants within one month in liquid medium with shaking at 100 rpm. (**F**) Final selection of putative transformants.

To determine the appropriate concentration of hygromycin for effective screening of transformed calli and plants, calli were cultured in medium containing different concentrations of hygromycin. Figure 3 shows the survival rate of calli after one month. With 50 mg/L hygromycin, the survival rate was zero, and the highest survival rate was observed with 10 mg/L hygromycin. To reduce the selection of non-transformants, i.e., for more accurate selection of transformed calli, we selected 20 mg/L hygromycin as the optimal concentration for selection (Figure 4A,C,F). During the selection process, calli were inoculated into selection medium for one month to allow transformed cells to develop into new calli while others died. Figure 4B shows well-grown calli on non-selection medium. The duration of plant selection was only seven days, and after seven days, healthy growing plants were transferred to fresh hygromycin-free medium to allow further growth for subsequent DNA extraction and analysis.

### 3.4. Effect of Different Growth Conditions on Callus Growth after Selection

**Figure 5:**
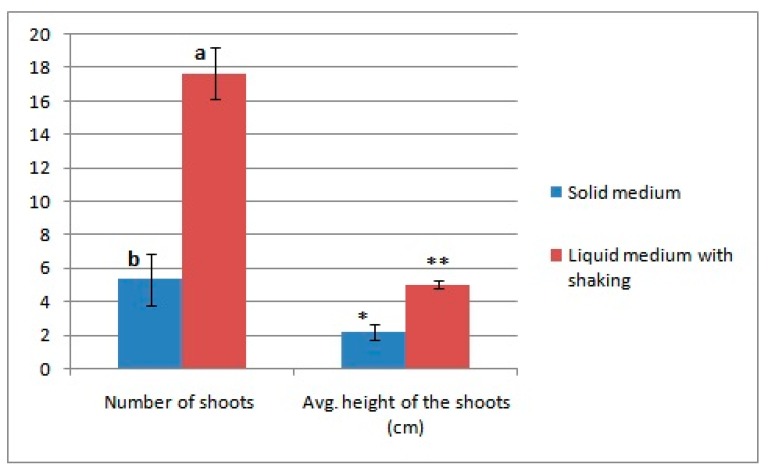
Effect of liquid medium with shaking on callus growth after selection. Error bars indicate standard deviation (SD). MINITAB 16 was used to perform ANOVA. Grouping information based on Fisher’s method. Different letters and different number of * represent statistically significant differences at *p* = 0.05.

With the goal of reducing the time required for the transformation process, we compared callus growth in liquid medium with shaking at 100 rpm and callus growth on solid medium two weeks after inoculation after the third selection. As shown in Figure 4D,E, a liquid medium with shaking led to better growth in terms of shoot number and shoot height (Figure 5). Therefore, a liquid medium could significantly reduce the time required for the transformation process from 7.6 months to 6.1 months (Table 4).

### 3.5. PCR Analysis

**Figure 6:**
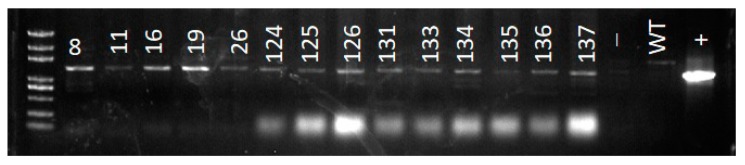
PCR confirmation of the *GFP-WRKY28* fragment in transformed plants.

To verify the presence of the transgenic construct in the genome of putative transformed plants selected through hygromycin resistance, genomic DNA was extracted using a modified CTAB method followed by PCR using specific primers for GFP and *AcWRKY28*. The expected *p35S:GFP-AcWRKY28* fragment was detected (Figure 6), indicating the presence of the transgene in the tested plants.

### 3.6. GFP-AcWRKY28 Is Localized in the Nucleus

**Figure 7:**
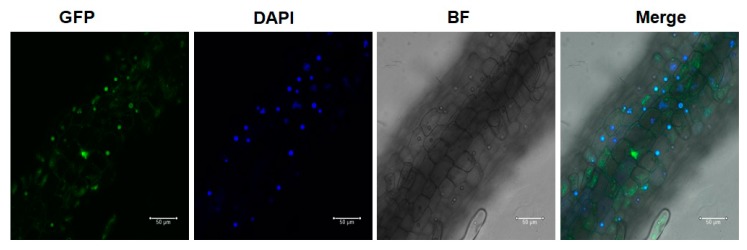
Confocal microscopy images of GFP expression in the nuclei of root cells in *p35S:GFP-WRKY28* plants. Bar = 50 µM.

*AcWRKY28* is predicted to encode a nuclear-localized protein, so we examined the localization of GFP-tagged AcWRKY28 [13]. Confocal microscopy and DAPI nuclear staining indicated nuclear localization of the GFP expression (Figure 7). These results support the nuclear localization of AcWRKY28 predicted by Xie et al. (2018) and show that our *Agrobacterium*-mediated transformation of pineapple with a GFP-tagged transgene was successful.

## 4. Discussion

*Agrobacterium*-mediated pineapple transformation has not been widely used for pineapple improvement efforts, due to the prolonged duration and technical difficulties of the transformation process. Despite the time and labor requirements of the process, however, a few studies have successfully introduced high quality agronomic traits into pineapples, such as delayed flowering through silencing of the ACC synthase gene, herbicide tolerance, and blackheart disease control [4,5,6]. Considering the importance of the crop, there is still much potential for pineapple improvement; in this context, pineapple self-incompatibility and its long life span continue to hinder traditional breeding efforts in pineapple. The studies that have been conducted have relied on herbicide tolerance genes or GUS as the marker genes to select positive plants. The GUS, luciferase, and anthocyanin reporter genes have been widely used in plant genetic transformation, with GUS being the most commonly used reporter gene. However, the detection of GUS is indirect and destructive [15], and expression localization at the organelle level is not possible when using GUS as the reporter gene, due to diffusion of color and require chemical treatment to observe desired expression [9]. In contrast, no exogenous substrate or cofactor required to form fluorescent signal and easily visible with the use of GFP as a reporter gene. Furthermore, GFP is stable with minimum denaturation at normal conditions [10]. Therefore, all these properties of GFP reporter gene make it an ideal non-destructive reporter gene, which can be used to tag localization of other genes easily. In the current study, we successfully introduced a 35S-driven GFP-tagged pineapple gene into pineapples. Compared to the GUS assay to observe positive transformants, the detection of GFP-tagged protein is non-destructive. Moreover, GFP tagged gene transformed plants can be used for the functional genomics, such as ChiP sequencing and ChiP qPCR. Therefore, the method we have developed can be used for the functional genomics analysis to help molecular breeding to improve desired traits. 

Successful and efficient transformation systems are also dependent on the efficiency and success of regeneration [18]. We have developed an efficient and cost-effective regeneration system for pineapple [19], which we used in the present study to obtain transformed plants.

There is some consensus that NPT-II may not be suitable for the transformation of legume crops and monocotyledons [18]. In our analysis, we used hygromycin resistance to select putative transformants during the regeneration process, similarly to previous related studies [7]. In addition to hygromycin, Basta (herbicide tolerance) and kanamycin have also been used for pineapple transformant selection in previous work [5,18]. 

Verification of stably and uniformly transformed pineapple lines could not be done using seed segregation analysis as it is typically done for inbred lines of seed-propagated plants [20]. However, we have confirmed the stability and uniformity of transformation using different parts of the plant such as roots and leaves. We used leaves to extract genomic DNA for PCR confirmation and roots to observe expression of the GFP-tagged protein.

In this study, we used embryonic callus for transformation to help minimize or eliminate undesirable somaclonal variation. We also reduced subculturing events to avoid potential somaclonal variation. We further optimized the bacterial infection time and hygromycin concentration for selection to improve transformation efficiency. During selection, we noticed that the callus regeneration ability was reduced in the selection medium. Therefore, after every round of selection, the remaining green calli were transferred to non-selection medium to promote growth.

*Agrobacterium*-mediated transformation is a basic plant biotechnology tool, but in many plant species and crops in particular, transformation efficiency has not yet reached a satisfactory level. Previous studies showed that ethylene negatively affects *Agrobacterium*-mediated transformation [21]. Specifically, increasing ethylene production by supplying its immediate precursor, 1-aminocyclopropane-1-carboxylic acid (ACC), suppresses transformation in tomatoes and melons [22,23]. Normally, ethylene levels increase in response to wounding and *Agrobacterium* infection during co-cultivation [23]. In pineapple transformation, the browning of calli during co-cultivation is a major drawback. This may be due to ethylene production, as observed in previous studies in other crops, or pineapple transformation efficiency might be reduced, due to ethylene production during co-cultivation. It is, therefore, necessary to study the effect of ethylene during co-cultivation of pineapple transformation and to develop strategies to overcome this hurdle to increase transformation efficiency. For example, super-*Agrobacterium*, which has ACC deaminase activity, may enhance T-DNA transfer into plants, and ethylene-absorbing chemicals, such as silver thiocyanate in the co-cultivation medium might help improve transformation efficiency in pineapple.

According to previous studies of the pineapple WRKY TF family, *AcWRKY28* (*Aco005719.1*) is an ortholog of *Arabidopsis AtWRKY51* [13]. *AtWRKY50* and *AtWRKY51* act as positive regulators of SA-mediated signaling and as negative regulators of JA-mediated signaling [14]. Therefore, our transgenic *AcWRKY28* over-expression pineapple lines may help unravel the molecular mechanism of this gene in pineapples in response to different stress conditions. 

With the successful transformation of GFP-tagged genes into pineapples through *Agrobacterium*-mediated transformation and the availability of improved regeneration methods, the introduction of useful agronomic traits may now be more achievable. The aforementioned suggested methods to eliminate ethylene biosynthesis during co-cultivation may help improve the transformation efficiency in pineapple. The present analysis shows that it is possible to introduce a GFP-tagged gene into pineapples, and to detect its expression in the transformed plants at the organelle level. These findings present a useful tool for basic research, functional genomics and genetic breeding of pineapple.

## Figures and Tables

**Table 1:** Different pineapple transformation media.

Media	Composition
Luria broth	Tryptophan 10 g, yeast extract 5 g, NaCl 10 g
AAM-AS (1 L) pH = 5.2	AA macro nutrient 10× (100 mL)–(KH_2_PO_4_-170 mg/L, MgSO_4_.7H_2_O-370 mg/L, KCl-2940 mg/L, CaCl_2_-440 mg/L) AA micro nutrient 100× (10 mL)–(MnSO_4_.4H_2_O-10 mg/L, H_3_BO_4_-3 mg/L, ZnSO_4_.7H_2_O-2 mg/L, KI-0.75 mg/L) AA micro nutrient 1000× (1 mL)–(NaMoO_4_.2H_2_O-0.25 mg/L, CuSO_4_.5H_2_O-0.0387 mg/L, CoCl_2_.6H_2_O-0.025 mg/L) Ferrous 100× (10 mL) AAM-AS organic 100× (10 mL)–(glycine-7.5 mg/L, L-arginine-174 mg/L, L-glutamine-876 mg/L, inositol-100 mg/L, nicotinic acid-0.5 mg/L, vitamin B1-0.5 mg/L, vitamin B6-0.5 mg/L, casamino acid-500 mg/L) Sugar 30 g, glucose 68.5 g, 200 µM acetosyringone
Co-cultivation medium	4.43 g/L MS powder, 30 g/L sugar, 3 g/L phytagel, 1 mg/L BAP, 0.2 mg/L NAA, 200 µmol/L acetosyringone pH = 5.8
Proliferation medium	4.43 g/L MS powder, 30 g/L sugar, 3 g/L phytagel, 1 mg/L BAP, 0.2 mg/L NAA, 200 mg/L carbenicillin pH = 5.8
Selection medium	4.43 g/L MS powder, 30 g/L sugar, 3 g/L phytagel, 1 mg/L BAP, 0.2 mg/L NAA, 200 mg/L carbenicillin, 20 mg/L hygromycin pH = 5.8
Rooting medium	4.43 g/L MS powder, 30 g/L sugar, 3 g/L phytagel, 0.2 mg/L NAA

**Table 2:** PCR mixture composition.

Reaction Component	Volume
Taq buffer	10 µL
Forward primer	0.5 µL
Reverse primer	0.5 µL
Sterilized distilled water	8 µL
Template DNA	1 µL
Total	20 µL

**Table 3:** Effect of incubation time on *Agrobacterium* infection.

Incubation Duration	Contaminated %	Death % before Selection	Growth %	Death % at 1st Selection
≤1 h	5 ± 0.0050 ^c^	1 ± 0.000 ^C^	84 ± 0.011 *	95 ± 0.000 ^d^
1 h ≥ *t* ≤ 6 h	7 ± 0.0100 ^c^	1 ± 0.000 ^C^	84 ± 0.010 *	79 ± 0.000 ^e^
8 h	24 ± 0.0115 ^b^	20 ± 0.020 ^B^	10 ± 0.000 **	79 ± 0.040 ^e^
12 h	71 ± 0.0153 ^a^	84 ± 0.040 ^A^	5 ± 0.000 ***	81 ± 0.026 ^e^

Grouping information based on Fisher’s method. Means that do not share the same letter are significantly different with a 95% confidence interval. Different letters and different number of * represent statistically significant differences.

**Table 4:** The time required to obtain transformed pineapple plantlets.

Transformation Step	Time (Days)
Transformation with *Agrobacterium*	1
Co-cultivation	3
Non-selection medium	7
First selection medium	28
Non-selection medium	28
Second selection medium	14
Non-selection medium	28
Third selection medium	14
Non-selection medium	56
Plant selection medium	7
Selected plants grown in rooting medium before transfer to soil	28
Total time	214 ≈ 7.6 months

## Abbreviations

TF	transcription factor
OD	Optical density
DNA	Deoxyribonucleic acid
GFP	Green flourascent protein
